# Optimizing Recovery: An Opportunity to Improve Access to Post-stroke Rehabilitation Care in Rural Settings

**DOI:** 10.7759/cureus.85939

**Published:** 2025-06-13

**Authors:** Kiera Murphy, Sarahrose Jonik, Andrew J Rothka, Neyha Cherin

**Affiliations:** 1 College of Medicine, Penn State College of Medicine, Hershey, USA; 2 Physical Medicine and Rehabilitation, Penn State Health - Milton S. Hershey Medical Center, Hershey, USA

**Keywords:** botulinum toxin injections, post-stroke spasticity, rural health, stroke, telehealth

## Abstract

Post-stroke spasticity (PSS) is a debilitating sequela that can lead to significant pain, severe functional decline, worse health outcomes, higher mortality rates, and increased healthcare costs. Botulinum toxin (BTX) injections are a widely recognized treatment modality to combat PSS. Not surprisingly, given that BTX administration requires a specialized provider and in-person visits, patients in rural communities are often unable to receive this vital intervention.

We present the case of a 59-year-old male who suffered a left ischemic thalamic stroke resulting in severe PSS. He was initially taken to a large academic center for his stroke care, followed by a two-week inpatient rehabilitation stay, during which he made significant progress. Unfortunately, once discharged to his rural community, he was lost to follow-up. Over time, he developed painful upper and lower extremity spastic hemiparesis, which impaired his ability to ambulate, complete independent activities of daily living (ADLs), and led to severe depression. Fortunately, the patient’s neighbor noted a significant decline in function and quality of life, prompting her to bring him to her Physical Medicine and Rehabilitation provider for possible intervention. Due to the kindness of his neighbor, the patient was able to reestablish care two hours away, allowing him to initiate BTX injections and address the unique challenges posed by his worsening spasticity.

In response to the patient’s rural residence, the authors developed a post-stroke telehealth follow-up protocol to ensure continuous virtual monitoring between in-person BTX injections. This case illustrates the potential of telemedicine to bridge the gap in care for patients residing in rural areas by leveraging the growing availability of internet access. We discuss the successful implementation of this telehealth follow-up protocol and propose it as a sustainable model for delivering essential care to underserved rural populations.

## Introduction

Stroke is the leading cause of long-term adult disability in the United States, affecting over 700,000 people annually [1]. Despite remarkable advances in research aimed at improving mortality outcomes, the comorbid complications from the initial neurologic insult - such as post-stroke spasticity (PSS) - continue to plague stroke survivors. According to the American Stroke Association, 25%-43% of stroke survivors experience PSS [2]. PSS commonly develops within the first three months post-stroke, with younger patients noted to be at higher risk [2]. When unchecked, uncontrolled spasticity can result in debilitating functional outcomes, severe pain, and impaired quality of life [3]. The scientific literature emphasizes early intervention, including botulinum toxin (BTX) injections, as paramount to minimizing the morbidity and mortality associated with PSS [4]. However, despite these advancements, translating research findings into real-world applications remains challenging - particularly for patients residing in rural areas. Existing disparities in rural healthcare access - such as provider shortages, limited access to specialists, and transportation barriers - exacerbate this gap, often leaving patients without timely or appropriate interventions. We hypothesize that vastly disproportionate access to standard medical care and resource availability, based on location of residence, is a major reason for this disconnect.

## Case presentation

We present a case of a 59-year-old male with a past medical history of hypertension and depression, who suffered a left ischemic thalamic stroke affecting the posterior limb of the internal capsule, resulting in severe, early-onset spasticity. Following his acute hospitalization, the patient underwent a two-week intensive inpatient rehabilitation stay, during which he made significant improvements in spasticity, range of motion (ROM), functional mobility, and activities of daily living (ADLs). The patient was discharged home with an ankle-foot orthosis (AFO), scheduled for home therapy follow-up, and referred to outpatient Physical Medicine and Rehabilitation (PM&R). Unfortunately, once discharged home to his rural residence, he was faced with limited access to healthcare services, including no local PM&R provider. Ultimately, he lost the functional gains he achieved during his rehabilitation stay. Additionally, his untreated spasticity progressed, resulting in severely painful right upper and lower extremity spastic hemiparesis. This led to an inability to utilize his AFO, which subsequently worsened his functionality and quality of life.

Fortunately, the patient’s neighbor was a patient of our PM&R clinic, and she recognized his declining function, worsening lethargy, and need for clinical care. After a discussion with the patient, he agreed to establish care in our clinic, despite the two-hour commute. Eleven months after his initial stroke, the patient presented to our PM&R clinic for evaluation. On initial examination, the Modified Ashworth Scale (MAS), a standard clinical tool that measures muscle tone, demonstrated significant spasticity in the right upper and lower extremities (Table 1).

**Table 1:** Comparison of MAS Scores Pre- and Post-treatment With BTX A larger number indicates increased resistance to motion. MAS: Modified Ashworth Scale; BTX: Botulinum Toxin

Muscle Group	Pre-treatment MAS Score	Post-BTX (300 Units) MAS Score
Shoulder Adduction	1	0
Elbow Flexion	1+	0
Elbow Extension	1+	0
Forearm Pronation	1	1
Wrist Flexion	2	1
Finger Flexion	2	1
Hip Flexion	2	1
Hip Extension	1	1
Dorsiflexion	1	0

A comprehensive treatment plan was initiated, including patient education on PSS, intervention with 300 units of BTX injections, physical and occupational therapy, and a new AFO for ambulatory support. Additionally, the patient expressed loneliness and isolation, along with signs of acute-on-chronic depression. While PSS management was the patient’s primary concern, we recognized the need to apply a multidisciplinary approach: social work was consulted, and the patient and his wife were referred to the hospital’s stroke survivor support group with video conferencing capabilities.

**Figure 1:**
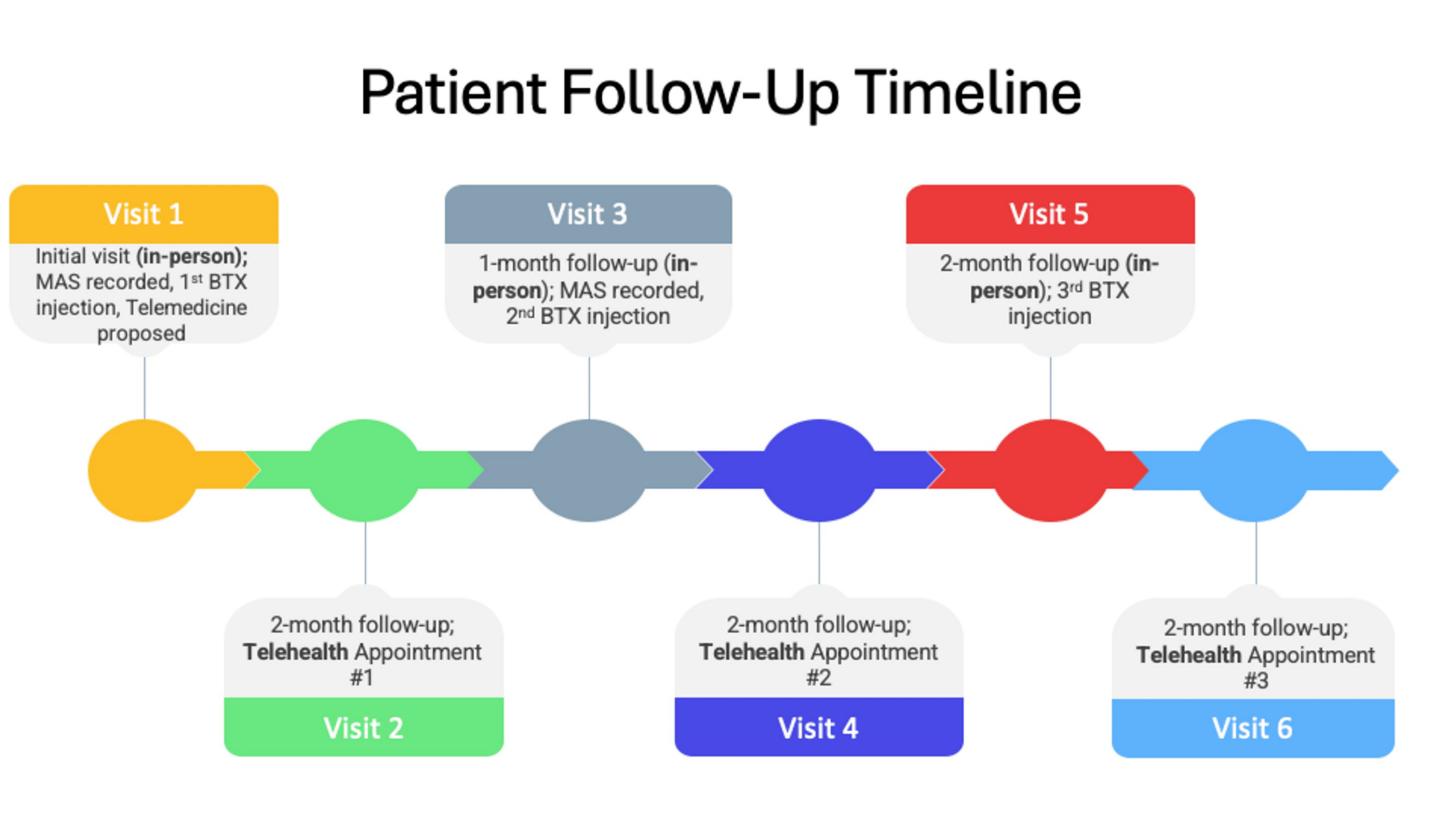
Patient Follow-Up Timeline MAS: Modified Ashworth Scale; BTX: Botulinum Toxin

Given the distance of the patient’s home to the clinic, a telehealth protocol was established for follow-up care in between BTX injections (Figure 1). Eight weeks following his initial BTX treatment, he was evaluated with PM&R via telehealth. At this visit, he noted significant improvements in all aspects of his care. While a MAS was not able to be completed via telehealth, the affected limb was able to be visualized and assessed subjectively for ROM. To assess ROM virtually, the patient was instructed to position their device to ensure clear visualization of the relevant body part. The patient was then verbally guided through a series of specific movements to evaluate symmetry and the extent of motion. Based on the findings from this assessment, adjustments to BTX dosing were considered for the upcoming visit. Additionally, the patient emphasized the substantial impact that the education had on his rehabilitation efforts. He began utilizing a daily home exercise program that focused on daily passive and active-assisted ROM, weight-bearing activities, and functional task practice. He also utilized his AFO along with nightly splinting to avoid muscle stiffness, established care with counseling services, and reflected in a gratitude journal to combat his worsening depression. The patient and his wife attended the virtual support group and noted that it had a positive influence on adjusting to life following a stroke; by connecting virtually with stroke survivors and their spouses, isolation was reduced, and a support network was established. 

The patient then returned three months post-initial injection for repeat BTX injections. At that in-person visit, the MAS was repeated, demonstrating marked improvement in ROM, stretching, exercise tolerance, tremors, pain control, and quality of life. Results of the MAS pre- and post-BTX intervention are provided in Table 1. Given the progress the patient was making with this treatment regimen, follow-up visits were scheduled at eight-week intervals, alternating between in-person and telehealth appointments to accommodate the patient's long travel distance to the clinic.

## Discussion

Stroke is the second leading cause of death in the United States [2]. For those who survive, PSS affects 25%-43% of patients, and its impact can be profound [2,5,6]. Treatment of PSS is often necessary and should be considered in situations where patients experience impaired ADLs and quality of life. Frequently, BTX injections are used in conjunction with other conservative modalities as first-line management for PSS [1]. BTX intervention can improve patient function by delaying contracture formation, increasing passive ROM, and aiding in orthotic use [7]. Indirectly, BTX injections can mitigate pain, improve hygiene maintenance, reduce caregiver burden, and avoid the systemic side effects of oral medications, especially in those with cognitive impairment [7]. While spasticity typically develops within the first three months post-stroke, BTX treatment is commonly not offered until six months post-stroke [1]. However, studies demonstrate that early initiation within the first three months has the greatest reduction in MAS scores and limb spasticity [1,7]. Furthermore, the ONTIME study found that BTX injections within 2-12 weeks post-stroke significantly increased the interval until re-injection criteria were met, delaying spasticity recurrence and consequently reducing healthcare costs [8].

Despite clear evidence supporting early intervention for PSS, many patients face delays in receiving this essential treatment. This gap consistently occurs at the informational level. To bridge this miscommunication, clinicians should educate patients and their families on the potential sequelae of comorbidities following a stroke. Also, engaging patients in discussions regarding goals of care, and involving family members in the decision-making process, can help bridge these communication gaps and improve patient outcomes [9]. Additionally, access to support groups for patients and their families has been found to improve quality of life in patients with PSS [10]. In our patient’s case, he noted significant improvements in his recovery once he received additional education in the PM&R clinic and was provided with social and medical support services.

In our patient’s case, through considering the state of his PSS and his response to therapy, virtual check-ins were established two months following BTX injections to allow for close monitoring of treatment response, as well as the opportunity for providers to understand how to modify the necessary dosage at future appointments. While prior studies have explored the utility of telehealth to monitor patients for modifiable risk factors and post-stroke complications, the strategy of specifically integrating telehealth in BTX management for PSS remains largely underexplored [11,12]. One recent study conducted by a team of Italian physiatrists examined this novel approach, including its benefits and challenges; they found that these virtual appointments had the added advantage of allowing providers to observe their patients in their home environment, providing them the opportunity to witness any functional limitations they may be experiencing within their daily life [13]. Additionally, virtual follow-up care in between BTX injections reduced the burden of frequent clinical visits for patients with impaired mobility [13]. They note that, while 73.3% of remote follow-up assessments were conducted successfully in their study, the use of telemedicine in spasticity management remains poorly documented and understood, and additional studies are needed to explore implementation challenges and long-term outcomes [13].

One key aspect of the delay in seeking treatment for our patient was his rural residence. Patients in remote areas are frequently without equitable access to resources, which can hinder treatment. Data support this, as stroke survivors living in rural communities exhibit educational deficiencies regarding follow-up care and disease management, resulting in worse health outcomes, higher mortality, and increased healthcare expenditure [14]. Over the past two decades, however, patients with lower socioeconomic status and those living in rural settings have gained increased access to the internet, making telehealth a more viable option [15]. While treatments such as BTX injections, requiring frequent in-person follow-up, may have previously been unfeasible for these patients, the growing availability of telehealth services is changing this dynamic.

While preparing patients for discharge, it is crucial to consider the setting of their residence. Options such as telehealth should be engaged to ensure needs are continuously met following placement back in one’s home environment, and a virtual telehealth follow-up schedule can be established based on the severity of symptoms to allow for ongoing management in between in-person appointments. Additionally, as seen in our patient’s case, referrals for virtual social support groups can be made to address feelings of isolation and considerably improve the patient's quality of life.

## Conclusions

In conclusion, this case underscores the critical role of incorporating virtual telehealth follow-up as an adjunct to in-person BTX injections in the management of PSS, particularly for patients residing in rural or underserved areas. By implementing a telehealth follow-up protocol, we were able to effectively monitor treatment progress, adjust BTX dosages, and provide essential educational and emotional support, all of which contributed to significant improvements in the patient's functional outcomes and quality of life. This approach demonstrates the potential of telemedicine to bridge gaps in care, ensuring continuity and accessibility for patients who might otherwise face significant barriers to in-person treatment. The successful application of this telehealth follow-up protocol offers a model for other clinicians and healthcare systems to adopt, such as for treating patients with other chronic neurological conditions that require intermittent in-person care. Additionally, this protocol can be utilized across multiple medical disciplines for patients with chronic conditions but long commutes to their healthcare providers. Future research should further explore the implementation, challenges, and long-term efficacy of telehealth in BTX management for PSS, to solidify its role as a vital component of post-stroke care.

## References

[1] Rosales RL, Efendy F, Teleg ES (2016). Botulinum toxin as early intervention for spasticity after stroke or non-progressive brain lesion: a meta-analysis. J Neurol Sci.

[2] (2025). Let’s talk about spasticity after stroke. https://www.stroke.org/en/help-and-support/resource-library/lets-talk-about-stroke/spasticity.

[3] Chohan SA, Venkatesh PK, How CH (2019). Long-term complications of stroke and secondary prevention: an overview for primary care physicians. Singapore Med J.

[4] Picelli A, Santamato A, Cosma M (2021). Early botulinum toxin type A injection for post-stroke spasticity: a longitudinal cohort study. Toxins (Basel).

[5] Francisco GE, Wissel J, Platz T, Li S (2021). Post-stroke spasticity. Clinical Pathways in Stroke Rehabilitation: Evidence-Based Clinical Practice Recommendations.

[6] Wissel J, Ri S, Kivi A (2023). Early versus late injections of botulinumtoxin type A in post-stroke spastic movement disorder: a literature review. Toxicon.

[7] Lindsay C, Ispoglou S, Helliwell B, Hicklin D, Sturman S, Pandyan A (2021). Can the early use of botulinum toxin in post stroke spasticity reduce contracture development? A randomised controlled trial. Clin Rehabil.

[8] Rosales RL, Balcaitiene J, Berard H (2018). Early abobotulinumtoxinA (Dysport(®)) in post-stroke adult upper limb spasticity: ONTIME pilot study. Toxins (Basel).

[9] Sunnerhagen KS, Francisco GE (2013). Enhancing patient-provider communication for long-term post-stroke spasticity management. Acta Neurol Scand.

[10] Bavikatte G, Subramanian G, Ashford S, Allison R, Hicklin D (2021). Early identification, intervention and management of post-stroke spasticity: expert consensus recommendations. J Cent Nerv Syst Dis.

[11] Naqvi IA, Cheung YK, Strobino K (2022). TASC (Telehealth After Stroke Care): a study protocol for a randomized controlled feasibility trial of telehealth-enabled multidisciplinary stroke care in an underserved urban setting. Pilot Feasibility Stud.

[12] Sharrief AZ, Guzik AK, Jones E, Okpala M, Love MF, Ranasinghe TI, Bushnell C (2023). Telehealth trials to address health equity in stroke survivors. Stroke.

[13] Spina S, Facciorusso S, Cinone N (2024). Integrating telemedicine in botulinum toxin type-A treatment for spasticity management: perspectives and challenges from Italian healthcare professionals. Toxins (Basel).

[14] Kitzman P, Hudson K, Sylvia V, Feltner F, Lovins J (2017). Care coordination for community transitions for individuals post-stroke returning to low-resource rural communities. J Community Health.

[15] Jensen JD, King AJ, Davis LA, Guntzviller LM (2010). Utilization of internet technology by low-income adults: the role of health literacy, health numeracy, and computer assistance. J Aging Health.

